# State of the Art Review of Cell Therapy in the Treatment of Lung Disease, and the Potential for Aerosol Delivery

**DOI:** 10.3390/ijms21176435

**Published:** 2020-09-03

**Authors:** Hosanna Brave, Ronan MacLoughlin

**Affiliations:** 1 College of Medicine, Nursing & Health Sciences, National University of Ireland, H91 TK33 Galway, Ireland; h.brave1@nuigalway.ie; 2 Department of Chemistry, Royal College of Surgeons in Ireland, D02 YN77 Dublin, Ireland; 3 School of Pharmacy and Pharmaceutical Sciences, Trinity College, D02 PN40 Dublin, Ireland; 4 Aerogen Ltd. Galway Business Park, H91 HE94 Galway, Ireland

**Keywords:** mesenchymal stem cell, conditioned media, aerosol, respiratory, ATMP, exosomes, secretome, lung, nebulizer

## Abstract

Respiratory and pulmonary diseases are among the leading causes of death globally. Despite tremendous advancements, there are no effective pharmacological therapies capable of curing diseases such as COPD (chronic obstructive pulmonary disease), ARDS (acute respiratory distress syndrome), and COVID-19. Novel and innovative therapies such as advanced therapy medicinal products (ATMPs) are still in early development. However, they have exhibited significant potential preclinically and clinically. There are several longitudinal studies published, primarily focusing on the use of cell therapies for respiratory diseases due to their anti-inflammatory and reparative properties, thereby hinting that they have the capability of reducing mortality and improving the quality of life for patients. The primary objective of this paper is to set out a state of the art review on the use of aerosolized MSCs and their potential to treat these incurable diseases. This review will examine selected respiratory and pulmonary diseases, present an overview of the therapeutic potential of cell therapy and finally provide insight into potential routes of administration, with a focus on aerosol-mediated ATMP delivery.

## 1. Introduction

**Figure 1:**
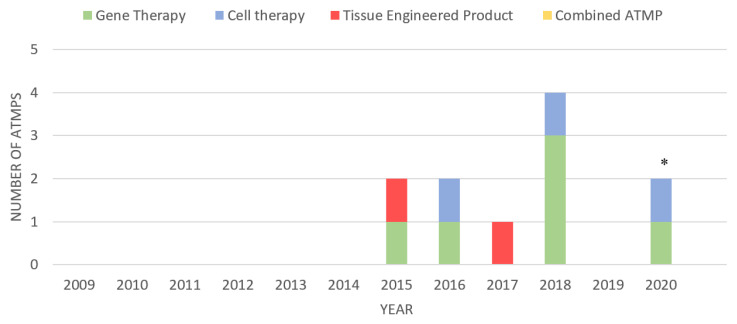
ATMPs (advanced therapy medicinal products; gene therapies, cell therapies, tissue-engineered products and combined ATMPs) that have been granted marketing approval by the EMA. Note: * indicates cell therapy “KTE-X19” expected to receive market authorization in 2020 (the EMA has validated their application but it currently under review [8,9]).

Advanced therapy medicinal products (ATMPs) are biological therapeutics primarily used for humans and animals. ATMPs can be classified into four types: gene therapy, somatic cell therapy, tissue-engineered products, or a combination of all three [1]. ATMPs are researched and developed in hospitals, academia, and small-sized enterprises [2]. Large pharmaceutical companies are not involved in the development and manufacturing of ATMPs since ATMPs are not of great interest to them [3]. Many of these ATMPs are still in the developmental stages [4]. ATMPs are said to bring about better health benefits, which include treatments for untreatable diseases and prevalent conditions [5,6]. There are a limited number of ATMPs that the European Medicines Agency (EMA) has approved (see Figure 1). Moreover, there are a profound amount of ATMPs being developed clinically. Plus, the number of ATMPs that are approved and licensed increases every year. Regulatory bodies such as the EMA give a positive and expert opinion on developed ATMPs which are then subsequently authorized by the European Commission (EC) [7]. Some ATMPs that have been approved and authorized include: Strimvelis^®^, Yescarta^®^, Imlygic^®^, Kymriah^®^, Luxturna^®^, and Zolgensma^®^ which are gene therapy medicinal products; somatic cell therapy products, Zalmoxis^®^, Alofisel^®^, and KTE-X19^®^; Holoclar^®^ and Spherox^®^, tissue-engineered products (see Figure 1). This present review will focus on cell therapies and their potential in treating various pulmonary and respiratory diseases and infections.

### 1.1. Stem Cells

Stem cells are non-specialized cells found in multicellular organisms and are capable of differentiating into more than 200 types of cell [10]. Their ability to self-renew and their excessive proliferative properties have drawn the attention of researchers and present significant therapeutic potential applications in the lung [11,12]. Stem cells can be used in several applications; stem cells can be isolated and expanded from human tissue and used for the replacement of damaged or injured tissue and the development of personalized medicine. They can also be used for stimulation of injured tissue, investigation of human development, and to broaden the knowledge of complex pathologies of diseases [13]. There are several types of stem cells, embryonic stem cells, adult stem cells (non-embryonic stem cells), hematopoietic stem cells, adipose-derived stem cells, umbilical/derived stem cells, cord blood stem cells, and amniotic fluid stem cells. Stem cells are found in both embryonic and adult cells [14]. Embryonic stem cells are found in the inner mass of blastocytes [15]. There has been a lot of interest in using these cells in repair and tissue regeneration. However, due to the safety and obvious ethical issues that revolve around the use of these cells, most researchers are inclined to use adult stem cells, notably mesenchymal stem cells (MSCs). MSCs also have the added advantages of low immunogenicity, minimized risk of teratoma formation, and high expansion potential ex vivo [14,16,17].

### 1.2. Mesenchymal Stem Cells

**Figure 2:**
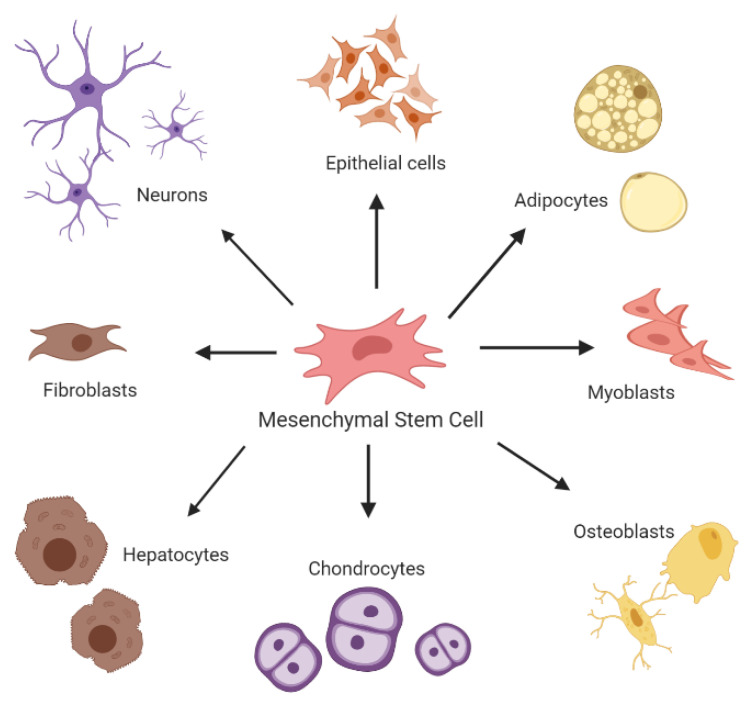
Overview of in-vitro differentiation of MSCs (mesenchymal stromal cells). Note: Figure created with BioRender.com.

MSCs, also known as mesenchymal stromal cells, are multipotent adult stem cells [18]. They are non-hematopoietic, tissue adherent stromal cells, and can be harvested from the bone marrow, cord blood, peripheral blood, fallopian tube, fetal liver, and lung. They are capable of self-renewal without the loss of their characteristics and have the capability of differentiating into different mesodermic and non-mesodermic tissue lineages (see Figure 2) [19]. The international society of cell therapy (ISCT) has set out several requirements regarding the characteristics of MSCs. They must be plastic adherent; they must express CD73, CD90, and CD105 and be absent of CD15, CD34, CD45, CD11, CD-79, CD-19 and HLA-DR. Lastly, under appropriate and specific conditions, they must be able to differentiate into chondrocytes, adipocytes, and osteoblasts [20].

**Figure 3:**
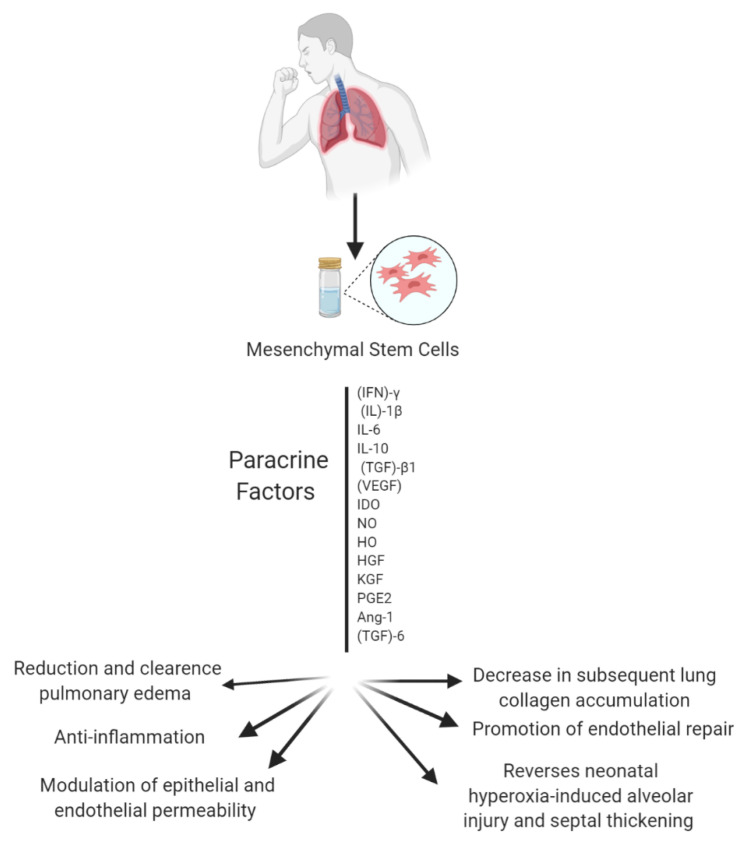
Overview of the several paracrine factors secreted by MSCs that can attenuate lung injury. Note: Figure created with BioRender.com.

MSCs have attracted considerable attention in the last thirty years; they have been studied extensively due to their potential application in medicine and research. MSCs are among the most widely used, well-known and well-documented types of stem cells [21,22]. MSCs’ remarkable anti-inflammatory, immunomodulatory, angiogenic, antifibrotic, anti-microbial properties are what has driven the interest in their application in regenerative medicine [23]. Interestingly, MSCs are also known to secrete painkiller molecules [24]. MSCs can reprogram the immune response and reduce the production of pro-inflammatory cytokines. MSC effects are based on their ability to home, engraft, and survive in the injured part of a lung. MSCs subsequently secrete paracrine factors, which in turn repair lung injury. A few paracrine factors that attenuate lung injury are IL-10, hepatocyte growth factor (HGF), and indoleamine 2,3-dioxygenase (IDO) [25]. MSCs’ paracrine factors combat factors that promote inflammation, apoptosis, and pathological remodeling (see Figure 3) [26].

### 1.3. Preconditioning and Potentiation of MSCs

In some instances, MSCs have been demonstrated to not be as effective in harsh environments, such as apoptosis and grave pathophysiological conditions [27]. Prior to administration, in preconditioned MSCs, notably, hypoxic conditioning has not only demonstrated the ability to enhance their functions in vitro and in vivo and protect them from the harmful milieu, but it can also improve homing ability and increase differentiation efficacy [27,28]. Collectively, this ameliorates their chances of survival in ischemic conditions. Preconditioning of MSCs entails the exposure of MSCs to unfavorable and stressful conditions such as hyperoxia, hypoxia, hydrogen peroxide, or stimulation of MSCs using various serums and agents such as heat shock proteins (HSP), melatonin or low doses of lipopolysaccharide (LPS) [28,29]. Studies have demonstrated that the use of HSP can protect MSCs against hypoxic-induced apoptosis [30]. Despite the considerable promise of the preconditioning of MSCs, the oxygen level, incubation time, and underlying mechanisms are still yet to be established [28]. Hypoxic conditioning can improve the expression of anti-apoptotic proteins amid ischemic conditions such as gas exchange impairment in ARDS (acute respiratory distress syndrome) [31,32]. To determine the effects of hypoxic preconditioning, Jiang et al. exposed human gingiva derived MSCs to hypoxic conditions of 2% oxygen. They demonstrated that the hypoxia increased the IL-10 production and the expression of FAS-L, which ultimately enhanced peripheral blood mononuclear cell (PBMC) proliferation and induced PBMC apoptosis [33]. Additionally, in a study carried out by Liu et al., hypoxic MSCs reduced the expression levels of the pro-inflammatory cytokines TNF-α, IL-10, and MIP-2, which, in turn, improved the overall lung function in acute ischemia/reperfusion (I/R) lung injury [34]. These two studies demonstrate that the preconditioning of MSCs can enhance their functions in ischemic conditions. Overall, it can enhance their therapeutic benefits in treating lung injury and disease.

### 1.4. Conditioned Media (CM)

Conditioned media is media enriched with a complex mixture of secreted products. Even though its mechanism of action is not yet fully understood, conditioned media has, in some cases, been shown to be better than cell therapies for ischemic and interstitial diseases [35]. Some of the beneficial secreted products by MSCs include cytokines, growth factors, extracellular matrix (ECM) protein, factors in matrix remodeling, and extracellular vesicles [36,37]. Research carried out thus far has shown that conditioned media has the ability to influence cell subtypes from the innate and adaptive immune system. A study carried out by Kim Yong demonstrated that MSC-CM led to the angiogenesis and regeneration of lung damaged by cigarette smoke by inhibiting cell apoptosis, inducing cell proliferation, and restored the expression of ECM proteins [38]. In another study carried out by Hwang et al., MSC-CM media was used to reduce the number of inflammatory cells that could migrate to lung tissue. [37]. A study carried out by Sagaradze et al. demonstrated that MSC-CM reduced the level of pro-inflammatory cytokine production, decreased infiltrating inflammatory cells. Furthermore, there was an increase in T cell subsets and macrophages. Sargaradze commented that even though these results have shown great promise, there is a lack of standards regulating bioprocessing and a lack of the use of auxiliary materials [35]. Additionally, in a study which set out to determine the protective effects of MSC secretome on pulmonary epithelial cells injured by hypoxia, Shologu et al. demonstrated that the pre-treatment of MSC-CM restored the matrix metalloproteinase (MMPs) physiological function in primary alveolar epithelial cells (AECs) that were subjected 0.5–1.5% O_2_ hypoxia. This ultimately promoted repair and cellular homeostasis of injured cells [39].

### 1.5. MSC Secretome

Secretome is a term used to define the set of proteins secreted by an organism to the extracellular space [40,41]. Secretome is specific and can alter in response to changes in physiological and pathological states and conditions [41]. There are a wide variety of immunomodulatory cytokines and chemokines secreted by MSCs such as growth factors, ECM proteases, hormones, and lipid mediators. Collectively these cytokines are known as MSC-secretome [42]. The MSC secretome has the capability of facilitating tissue regeneration, tissue repair, cytoprotection, and angiogenesis [41]. It is also capable of combating processes and factors from diseases such as apoptosis, pro-inflammatory cytokines, and pathological remodeling [42]. Of note, and important in the reliable and reproducible generation of MSC secretome, there is a lack of codified standardized preconditioning methods and the effects that preconditioning could potentially have on the MSC secretome. Nevertheless, and with this variability inherent, one of their main advantages is that they are secreted continuously by MSCs meaning there is less of a need for larger quantities of MSCs for production and treatment which combats the issue of escalating donor shortages [43,44]. Furthermore, the MSC secretome offers additional advantages over MSCs, such as complete avoidance of tumorigenicity and emboli formation. The MSC secretome can be administered and researched without the need for extensive expansion methods, a key enabler in their potential as a therapeutic approach [41,45].

### 1.6. Extracellular Vesicles (EVs)

Vesicles can be found in cell culture supernatants, blood, urine, synovial fluid, sputum, pleural effusion, and the alveolar space [44]. The immunosuppressive, protection, and reparative properties of EVs that are isolated from MSC supernatants are similar to MSCs themselves. There are three types of EVs: exosomes, microvesicles, and apoptotic bodies. EVs are categorized on the basis of their origin, size, and mechanism of biogenesis [46,47]. EVs and exosomes are produced from various activities that take place in the cells, while apoptotic bodies are secreted during apoptosis [47]. Natural EVs can deliver therapeutic agents to the desired site. EVs also have several more advantages compared to MSCs. EVs can withstand temperatures as low as −80 °C without losing their bioactivity benefits. EVs do not have the ability to self-replicate, so there is a complete avoidance in the formation of tumorigenicity and emboli formation [41,43]. In relation to respiratory diseases, EVs have shown their unique benefits by ameliorating alveolar specification, correcting pulmonary hypertension, and reducing the activating macrophage and pro-inflammatory cytokine levels [48].

### 1.7. Exosomes

Exosomes have been demonstrated to contribute to the therapeutic effects of MSC secretome [49]. Exosomes are nano-sized, non-self-renewing extracellular vesicles that originate in the endopathic pathway. They are then subsequently secreted by MSCs, T-cells, dendritic cells, tumor cells, and mast cells when multivesicular bodies (MVBs) and the plasma membrane fuse together [50,51]. The composition of the exosomal proteome includes cytoplasmic protein, nucleoproteins, and intracellular plasma membrane. Exosomes are involved in several vital roles in biological signaling and cell–cell signaling mediation [50,52]. They are also known to target housekeeping biological processes that operate within various tissues [53]. Exosomes provide several advantages compared to MSCs; they are non-replicating and can be sterilized by filtration due to them being nano-sized [54].

### 1.8. Cell Therapies

**Figure 4:**
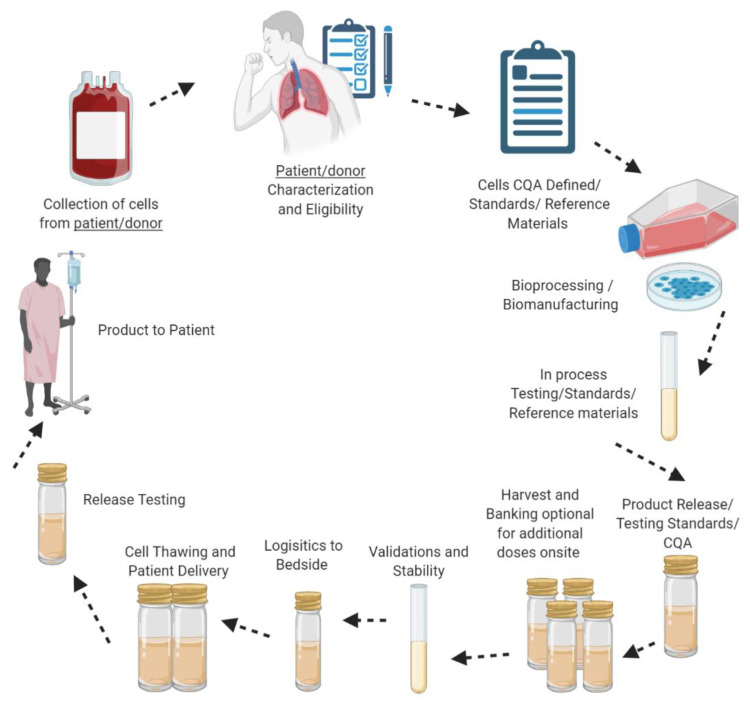
Overview of the production/manufacturing process of autologous and allogeneic cell therapy approaches. Note: Figure created with BioRender.com.

Cell therapy can be defined as a therapeutic application of cellular material despite the type of cell or clinical indication that can be administered to an animal or human [55,56]. The primary aim of this type of biotherapy is to develop medicinal products and therapeutic strategies to repair and stimulate damaged tissue and organs such as myocardial regeneration, skeletal muscle, and lung tissue [57]. Furthermore, the aim is to ultimately cure diseases and conditions such as lung diseases, neurodegenerative disorders, spinal cord injuries, cancers such as leukemia and melanoma and autoimmune diseases such as diabetes, multiple sclerosis, and rheumatoid arthritis [57,58,59]. Chemically produced drugs and cell therapy differ in the sense that cellular therapies can be personalized to cure a disease unlike the majority of organic compounds that are developed to suppress symptoms and not to cure a disease [55]. There are two conventional cell therapy approaches: Autologous, patient-specific (the harvesting and expanding of cells from an individual and reintroducing them back into the same individual) [60], and allogeneic (patient independent), cells from a donor that are isolated and expanded and reintroduced back into a recipient (see Figure 4) [57,60,61]. In terms of MSCs, studies have demonstrated that autologous cell therapy is a more potent and effective strategy compared to allogenic [62]. In addition, allogenic cell therapy also has its limitations, notably the possibility of immune rejection [63]. However, the choice between the two strategies depends on the circumstance at hand. Preclinical trials have demonstrated that autologous cell therapy may not be the best option for chronic lung disease [64]. It may be challenging to isolate and expand cells from older patients due to their decreased biological activity, or from a patient that is severely ill due to their immune suppression properties and low immunogenicity, and the isolation and expansion of MSCs is a time-consuming process [65]. In terms of lung diseases, and depending on the patient’s status, there may not be enough time to isolate and expand the cells of the patient. An off the shelf allogeneic therapeutic strategy would be a viable option for chronic lung diseases.

Cell therapies have advanced in the last decade. According to the EMA, there are currently four cell therapies that have been approved for marketing in Europe (See Figure 1). They have garnered significant interest due to their characteristics, long term management, and potential to treat untreatable diseases, however, there are still safety, toxicity and tumorigenicity issues that revolve around the use of pluripotent cellular therapies [66]. To maximize the potential benefits of cell therapies, it is imperative that there are more established strategies and more research on the differentiation and paracrine mechanisms of stem cells and how they integrate and migrate in the body [55]. Nevertheless, with the research and preclinical and clinical studies done thus far, cell therapies and their regenerative abilities will very likely be a valuable asset in the therapeutic arsenal.

## 2. Pulmonary and Respiratory Diseases and Infections

Lung diseases are common conditions worldwide. They are broadly segmented into those that affect the airways and those that affect the alveoli. The diseases that affect the airways include asthma, chronic obstructive pulmonary disease (COPD), chronic bronchitis, emphysema, acute bronchitis, and cystic fibrosis. The diseases that affect the alveoli include pneumonia, tuberculosis, emphysema, pulmonary edema, lung cancer, acute respiratory distress syndrome, and pneumoconiosis [67]. Cell therapies have been at the forefront of respiratory and pulmonary diseases and infection in the last decade. This review focuses on chronic lung diseases only given that the bulk of the published literature focuses on those, as well as being supported by currently registered clinical trials. Specifically, we shall discuss the potential benefits of cell therapy in the treatment of ARDS, sepsis, and COVID-19.

### 2.1. Acute Respiratory Distress Syndrome (ARDS)

ARDS can be defined as an acute inflammatory life-threatening disease, which results in pulmonary gas exchange failure and ultimately causes the lungs to endure grave injury [68,69,70]. ARDS can arise from various indirect and direct consequences: direct lung injury, including pneumonia, aspiration of gastric contents and near-drowning and indirect lung injury including sepsis, severe trauma, drug overdose, pancreatitis, and transfusion injury [71]. ARDS can also be the result of intricate interactions with several inflammatory mediators such as TNF-α, IL-10, TGF-β and IL-6 [72], which result in diffuse alveolar damage, increased intrapulmonary stunts, increased capillary permeability, damage to the capillary endothelium non-cardiogenic pulmonary edema, and surfactant dysfunction [73]. These consequences correlate to the impairment of fluid removal from the alveolar space, which contributes to the accumulation of protein-rich fluid in alveolar, which results in the diffusion of damage in the alveoli [74,75]. Mild, moderate, and severe cases of ARDS are based on the severity of symptoms, the PaO_2_/FiO_2_ ratio, and the level of hypoxemia [70]. The PaO_2_/FiO_2_ ratio is used to indicate the oxygenation status of a patient in the intensive care unit (ICU) and to identify the hypoxemic respiratory failure when supplemental oxygen is being administered to a patient [76]. Mechanical ventilation and other inhalation/respiratory dosage forms are administered to improve tissue oxygenation but may sometimes cause pulmonary injury [74]. ARDS is present in 10% of patients in the ICU and remains a significant issue. [71,77]. The mortality rate of severe ARDS is approximately 45% [73]. As of now, no pharmacotherapy exists for the pathophysiological mechanisms of ARDS [78,79]. However, ARDS is commonly managed by mechanical ventilation and fluid conservation [71].

### 2.2. Sepsis

Sepsis is a critical and life-threatening medical condition for which no cure currently exists [68,80]. In January 2020, the University of British Columbia stated that sepsis is the leading cause of death globally [81]. Sepsis is also the cause of 50% of all inpatient deaths and arises when the body has been dramatically affected by bacterial, viral, or fungi infection(s) such as pneumonia, and the consequent inflammation and organ dysfunction [82]. Sepsis is also known as a variant of ARDS. The lung is the most affected organ due to pneumonia being the key starting point of the septic process [83]. Sepsis can affect gas exchange and pulmonary hemodynamic alterations because of increased capillary permeability and pulmonary pressure in the first stages of sepsis [84]. Early symptoms of sepsis include fever, shock, and multi-organ failure as well as hyperinflammatory innate immune responses [85]. Severe sepsis can cause failure to vital organs such as the lungs, kidneys, and liver [68]. Over the years, treatments for sepsis have immensely improved. Treatments and strategies including lung-protective ventilation, prone positioning, extracorporeal membrane oxygenation, fluid resuscitation, antibiotics, anti-microbial therapy, and blood products [82,83]. However, sepsis still has a high mortality rate [86].

### 2.3. COVID-19

Coronaviruses are enveloped non-segmented positive-sense RNA viruses that belong to the Coronaviridae family. The majority of human coronaviruses infections are mild [87]. By late December 2019 and early January 2020, it was noted that there were six human cases of novel COVs that had the capability to cause acute respiratory diseases and enteric and central nervous systems infectious and diseases in animals as well as humans: HCoV-229E, HCoV-OC43, HCoV-NL63, HKU1, SARS-CoV and MES-CoV [88,89]. Severe acute respiratory syndrome coronavirus (SARS-CoV) and Middle East respiratory syndrome coronavirus (MERS-CoV) are the two novel CoV infections that cause respiratory infections [88]. In December 2019, an outbreak of a novel coronavirus took place in Wuhan, China [90]. By January 2020, WHO (World Health Organization) declared the outbreak a public health emergency of international concern [91]. In February 2020, the novel virus was then isolated, identified, and named SARS-CoV-2 by the World Health Organization [90]. Since the initial detection of the virus, COVID-19 has infected over 25.3 million people and caused an estimated 848,000 deaths globally.

While the vast majority of individuals infected by coronavirus are thought to remain asymptomatic, the physical presentation of COVID-19 is dry cough, fever, chills, headaches, muscle pain, shortness of breath or difficulty breathing, sore throat, or loss of taste or smell [92]. Less common symptoms include sputum and hemoptysis [93]. Reports suggest that the elderly and those who have one or more underlying health conditions appear to have a higher risk of being infected by this disease [94]. As it stands, the medical observation period or quarantine period for people that have tested positive for COVID-19 is 14 days [95]. COVID-19 can cause pneumonia, ARDS, and sepsis, which can lead to long-lasting or permanent damage to the lungs, multiple organ death and, ultimately, death [96]. Medical disciplines have reported that patients that have pneumonia caused by coronavirus had alveoli filled with liquid (pulmonary edema), which can lead to the inability to take in oxygen. ARDS caused by coronavirus is the result of more air spaces becoming filled with fluid leaking from the blood vessels. In addition, COVID-19 patients diagnosed with ARDS lose the ability to breathe on their own [97,98].

**Figure 5:**
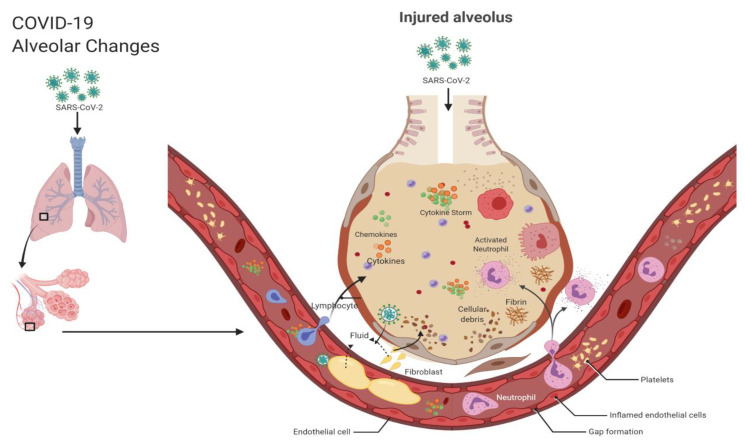
COVID-19 alveolar changes. Note: Figure created with BioRender.com.

Currently, the pathophysiology of COVID-19 is not entirely understood. Researchers have suggested that the coronavirus induces the development and implementation of several pro-inflammatory cytokines (IFN-γ, IL-1B, IL-6 and IL-12, 1L-2, IL-7, GSCF, IP10, MCP1, MIP1A, and TNFα) and pro-inflammatory chemokines (CXCL10 and CCL2). The cytokine and chemokines released subsequently initiate viral replication and infiltration [99,100]. However, it is the dysregulated activity of these cytokines and chemokines that results in the grave manifestations of COVID-19. In this paper, the term used to describe this phenomenon is “the cytokine storm”. The virus first travels down the respiratory tract and induces an innate immune response [101]. Inflammatory exudates and erythrocytes migrate into the alveoli which results in dyspnea and respiratory damage [102]. The cytokine storm results in alveolar damage with fibrin-rich hyaline membranes and a few multinucleated giant cells, epithelial cell proliferation, and increase of macrophages [101]. The debris from this damage then builds up and begins to cover the alveoli. The alveoli then become thicker than usual, impeding gaseous exchange [99,103]. The blood vessels around the alveoli weaken, which allows fluid to seep into the lung cavities, resulting in respiratory failure (see Figure 5).

The immune capacity of some antivirals is not strong enough to combat the cytokine cascade [104]. Antivirals, antibiotics, and biologics such as hydroxychloroquine, favipiravir, and tocilizumab have a significant effect on the underlying processes of COVID-19. However, the approach of this kind carries with it well-known limitations such as combating the cytokine storm. These pharmacological therapies can induce a range of adverse effects and outcomes which can cause morbidity and mortality [105]. Systemic corticosteroids can be effective but may reduce essential immune activity [106]. In addition, viruses, notably COVID-19, have the ability to suppress T cell functions which can make some of these therapies inefficacious [105].

## 3. Route of Administration

There are a variety of administration routes that can be used to administer a drug. The route of administration can either be enteral, parenteral, or topical [107]. The parenteral routes are intravenous (a drug given into a peripheral vein 1–2 min by infusion), intramuscular, subcutaneous, intra-arterial, and intradermal. Enteral dosage forms include oral, sublingual, buccal, or rectal. Intravenous (IV), intramuscular (IM), and subcutaneous (SC) are the most common parenteral routes [108]. The localization and persistence of MSCs after IV administration to the lung remains unknown. The route of administration is crucial due to the fact that it ultimately determines the fate of a drug, in this instance ATMPs. In some cases, standard drug delivery systems can be unsuccessful or ineffective because of their inability to deliver therapeutic agents to a target [109].

### 3.1. Intravenous Administration

The intravenous route is often the preferred route due to its ability to deliver large doses of medication to the body directly. Whilst not effective in delivering large doses of the therapeutic to the lung, some formulation strategies such as excipient choice, or even addition of lung-targeting ligands may increase drug concentration to a therapeutic level in the lung and can reduce its distribution and losses within other organs and tissues [109]. An advantage of using the intravenous route is its ease of application. However, there may be difficulty finding suitable veins, and associated tissue damage. The administration of MSCs via intravenous routes have been studied thoroughly. Of relevance here, MSCs that avoid first pass losses naturally migrate to the lung after administration and are usually trapped in the lung. MSCs then repair sites of injury via their immunomodulatory effects [110,111].

### 3.2. Intratracheal Instillation

Intratracheal instillation is the process by which liquid is introduced into a lung instantaneously via an intratracheal tube or microsprayer device. Instillation is not a physiological route for humans [112]. There are several advantages in that only a small amount of therapeutic is needed, and the doses can be precisely administered to the respiratory tract [113]. That said, intratracheal instillation may lead to inconsistent deposition, largely in the upper airways (heterogenous distribution), and medication tends to rely on a gravitational and regular mucociliary beat to disperse throughout the lung, thus leading to inconsistent deposition patterns [113]. Xisto’s comparative study in 2011 hypothesized that intratracheal administration was more effective than intravenous administration when modulating the inflammatory and fibrogenic “process” [114].

### 3.3. Inhalation

Inhalation therapy has been used for 1000 years [115]. In the last ten years, there have been significant advancements to inhalation drug therapies and new drug products for respiratory disease and fascinating neurological diseases [116]. Inhalation/aerosol drug delivery has played a significant role in many lung and respiratory diseases.

### 3.4. Aerosolization

Aerosols commonly consist of fine solids or liquid particles that are suspended in air or gas [117]. Aerosol therapy is generally used in critical pulmonary treatment [118]. There are several advantages that make aerosol drug delivery a preferred route of administration over various other types of dosage forms: It can be self-administered by patients, parents, and caregivers [119,120,121,122]. Various quantities of medication can be quickly released from a container without the risk of contamination or exposure to the remaining medicines [121,122]. The four most commonly used aerosol drug devices are nebulizers (NEB), soft mist inhalers (SMI), pressurized metered-dose inhalers (pMDI), and dry-powder inhalers (DPI) [123]. The development of pharmaceutical aerosol technology began with the MDI (metered-dose inhaler) [123]. The first MDI was reported in 1956 by Riker Laboratories Inc. [119,123].

### 3.5. Nebulizers

Nebulizers convert liquid substances into an aerosol. There are three types of nebulizers: jet nebulizers, mesh nebulizers and ultrasonic nebulizers. Nebulizers have the ability to deliver larger doses of medicines without the need for patient coordination [115]. MDIs are the most frequently used type of nebulizer and are used for various lung diseases such as severe asthma and chronic obstructive pulmonary disease. Even though nebulization therapy has many benefits, many nebulizers, such as jet nebulizers, have several limitations, poor drug solubility, and are unable to consistently deliver accurate and consistent medication [121]. The closed circuit, vibrating mesh type nebulizers do not have the same technological limitations, and may represent the most appropriate choice for ATMPs. Additionally, the majority of the current literature on aerosolized ATMPs makes use of vibrating mesh nebulizers. One of the main advantages of nebulizers is that they are capable of nebulizing a variety of therapeutics. Other benefits include the ability to modify the dosage amount administered to a patient [120].

## 4. Pre-Clinical Studies

### 4.1. ARDS

Recent studies have shown that cell therapies, specifically MSCs, MSC-CM, exosomes, and conditioned media, hold great promise in the treatment of ARDS [124]. MSC therapy is an exciting prospect because it can respond to the level and nature of an injury [74,75].

It has been debated that the lung has limited regenerative capacities [64,74]. However, as we have set out above, therapies such as MSC secretome, exosomes and conditioned media can be produced and stored and still maintain their viability prior to administration. Moreover, studies have demonstrated that cellular therapies consequently enhance cellular functions to regenerate circulating stem cells and attenuate survival when cells are administered directly to the bronchial tree in animal models [74,125]. In a study carried out by Maron-Gutierrez et al. a set of BALB/c mice were randomly administered saline or bone marrow-mesenchymal stem cells (BM-MSCs) intravenously. After day 1, there was a significant reduction in lung inflammation and increased remodeling, resulting in the improvement of lung mechanics in the extrapulmonary models [32]. In a separate study carried out by Cardenes et al., BM-MAPCs were administered to sheep to investigate the biodistribution of MAPCs for the treatment of ARDS. After 1 to 5 h, the cells had biodistributed to a variety of organs with the lung being the primary organ of retention. MAPCs also influenced arterial oxygenation recovery [126]. A study conducted by Gupta demonstrated that when MSCs were administered via an intrapulmonary route not only was there an improved survival rate of mice but they also reported downregulation of pro-inflammatory immune responses to endotoxin and also an increase in anti-inflammatory cytokines IL-10 [125]. Ionescu et al. demonstrated that MSC-CM led to the attenuation of lung inflammation and promotion of wound healing/anti-inflammatory M2 macrophage phenotype in part via IGF-1. This collectively results in alleviation of LPS-induced lung injury [127]. Similar to Ionescu et al., MSC-CM also attenuated LPS-induced ALI by the promotion of neutrophil apoptosis. Su et al. suggest that the treatment for LPS-induced ALI consists of the regulation of apoptosis in neutrophils, a crucial factor in alleviating LPS-induced inflammation [128]. Together, these studies suggest that MSC-CM could be a considerable alternative for treating ARDS.

### 4.2. Sepsis

There are no treatments that directly target the pathogenesis of dysregulated inflammation and tissue injury [129]. The pleiotropic effects of stem cells have the potential to intervene at various levels in the pathophysiology of sepsis in murine models, and subsequently reduce excessive inflammation which can prevent organ damage and could potentially reduce the rate of mortality [130]. Mesenchymal stem cell-based therapy has been shown to reduce severe bacterial sepsis in murine models because of their immunomodulatory and anti-microbial properties [131]. A study carried out by Gonzalez-Rey et al. showed that human or murine adipose-derived mesenchymal stem cells (AD-MSCs) in a sepsis model increased survival in mice [132]. In addition, stem cells have also demonstrated the ability to secrete growth factors in response to LPS and tumor necrosis factor that can result in the reduction of apoptosis and organ injury [133].

Of note, these results sometimes do not carry over in larger animal models. In a study carried out by Horak et al., one dose of BM-MSCs was administered to pigs. That group demonstrated that the infusion of MSCs was well tolerated, but the MSCs failed to ameliorate the septic conditions and failed to modulate the immune system’s inflammatory response [134]. This study is heavily contrasted with a study conducted by Laroye et al., where umbilical cord-mesenchymal stem cells (UC-MSCs) improved the survival of pigs by attenuating the hypotension, hyperlactatemia, and multiple organ failure [135]. Horak et al. suggests the immunomodulatory capacities of BM-MSCs and UC-MSCs may differ [134]. Furthermore, this discrepancy could be attributed to the limited ability of bone marrow-derived MSCs to secrete paracrine factors needed to attenuate septic pathophysiological conditions [136]. These findings, while preliminary, suggest that extensive research on a cell type and cell therapy type should be conducted prior to it being applied to a study. Furthermore, importantly, one should perhaps implement a preconditioning or potentiation process prior to BM-MSC administration to enhance their pleiotropic effects.

### 4.3. Aerosolization of Stem Cells In Vivo And In Vitro

The debate continues about the best strategies to administer MSCs, EVs, and MSC-CM for the treatment of respiratory and pulmonary diseases. There is also growing interest in the use of safe and more effective administration routes for respiratory disease treatment [137]. A limited amount of literature has been published on the effect of aerosolized stem cells and their benefits in treating respiratory diseases. Studies have demonstrated that nebulizers facilitate a high efficiency compared to various parenteral routes. Moreover, accumulated evidence and findings from preclinical studies investigating aerosolized MSCs supports the theory that inhalation and aerosolization of stem cells not only can lead to a high level of distribution of stem cells in the lung, but also stem cells maintain a high level of viability [83], and there is also a minimized risk of cell loss and morphological changes [138].

Aerosolized MSCs expressing angiopoietin-1 were investigated to demonstrate their effects in asthma-related airways in a rabbit. Their findings demonstrated that MSCs attenuated the airway inflammation and structural changes and also reduced the expression of various pro-inflammatory genes [138]. The use of aerosolized MSCs alone significantly reduced the levels of IL-4 and TGF-β. This study also compared aerosolized MSCs to non-aerosolized MSCs. The cell morphology of the aerosolized MSCs and the non-aerosolized MSCs demonstrated a significant difference between them. Furthermore, the aerosolized MSCs showed a high level of viability even though there was pressure on the MSCs during the aerosolization process [138]. Similarly, a study carried out in 2016 by Kim et al. demonstrated that atomized MSCs maintained a high level of viability [83]. In a comparative study by Averyanou et al., rabbits were administered MSCs via intravenous and inhalation routes. Five rabbits were administered 2 × 10^6^ MSCs via intravenous infusion. Their primary aim was to evaluate the survival rate of MSCs. This was demonstrated by using three types of nebulizers: jet, ultrasound, and mesh nebulizers. After 28 days, they assessed the morphological changes in bronchoalveolar lavage fluid (BALF). The jet nebulizer demonstrated the highest level of survival. It was also noted that there was evidence of significant antifibrotic effects on bleomycin-induced lung fibrosis [139].

The pioneering work carried out by Alhasan et al. investigated the viability of aerosolized MSCs under surface acoustic wave (SAW) nebulization. They demonstrated that SAW nebulization had no adverse effects on the cell metabolic rate, proliferation, or phenotypic characteristics compared to the non-nebulized MSCs [140]. A growing body of studies has investigated the concept of nebulized MSCs, MSC-CM, and MSC-secretome. MCS-CMs compatibility with vibrating mesh was first demonstrated by McCarthy et al., where MSC-CM inhibited the proliferation of pathogens via nebulization. This was demonstrated by nebulizing MSC-CM, collecting the aerosol and determining the antibacterial properties [141]. This result can be compared to a later study carried out Averyanov in 2018; that group demonstrated the compatibility of MSCs with three different types of nebulizers. In his study, the MSCs passed through the compressor nebulizer maintained a high level of viability followed by the ultrasonic of which only 20% of cells were viable and, surprisingly, there were no viable cells after they passed through a mesh nebulizer [142]. These findings indicate that MSCs may not be compatible with certain types of nebulizers; however, MSC-CM, MSC secretome, and exosomes may be compatible. Therefore, cell-based therapy compatibility with nebulizers will need extensive research prior this strategy being used in a clinical setting.

**Figure 6:**
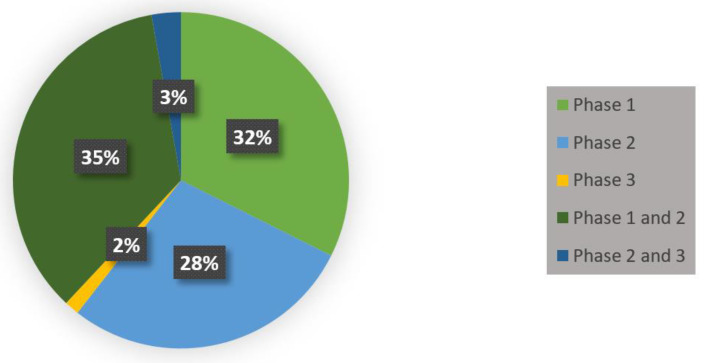
COVID-19, ARDS and sepsis MSC-based therapy-related clinical trials in phase 1, phase 1 and 2, phase 2, phase 2 and 3, and phase 3.

The evidence and findings presented in preclinical trials thus far have made MSC therapy attractive to clinical settings. A considerable amount of literature has been published on the potential benefits of MSCs. Every year millions of euros are invested in the planning and conducting of innovative clinical trials. These studies have shown that MSCs have a profound number of unique benefits and can be a promising treatment for pulmonary and respiratory diseases. To date, there are over 75 MSC-related clinical trials registered for ARDS, sepsis, and COVID-19 that are showing positive results (see Table 1, Table 2 and Table 3). A significant number of these clinical trials primarily focus on the safety and efficacy of administering MSCs (see Figure 6). However, several clinical trials lack results and publications on their findings. Furthermore, many of these trials mimic trials that have proven to be successful from findings published. It is clear that studies are beginning to evolve, and positive trends will emerge. The majority of clinical trials have demonstrated that the most common route of administration of MSCs is intravenous.

The majority of MSC-related clinical trials for ARDS are currently still in phase one and are focused on demonstrating the safety of intravenous administration of MSCs (see Table 1). In a clinical trial conducted by Wilson et al., human-derived BM-MSCs were not only safely administered to patients intravenously but also were tolerated well. Wilson et al. have since proceeded to phase 2, but the findings and outcomes have yet to be published [78]. Similarly, a clinical trial conducted by the University of California by Matthay et al. demonstrated the safety of one dose of MSCs to patients with severe ARDS. However, the viability of MSCs had to be improved [71]. To date, no clinical trials are using aerosolized MSCs. Therefore, future research should concentrate on the safety and efficacy of aerosolized MSCs, EVs, and MSC-CM, and examine their potential in a clinical setting.

Similar to cellular therapy-related clinical trials for ARDS, most cellular therapy-related trials for sepsis are currently in phase one demonstrating the safety and tolerability of MSC administration (see Table 2). In 2017, a published clinical trial by McIntyre et al. aimed to demonstrate the safety of administrating one dose of allogenic MSC to participants. They found that the infusion of 1 dose of MSCs “seemed” safe [143]. In a clinical trial conducted by Perlee et al., adipose-derived MSCs were intravenously administered to a human endotoxemia model. They (AD-MSCs) were well tolerated. The AD-MSCs also demonstrated time-dependent pro-inflammatory and anti-inflammatory effects [144]. However, several cellular therapy-related clinical trials have been deemed unsuccessful, even though many preclinical trials that administer MSCs to mice have been demonstrated to modulate inflammation, repair tissue, and enhance pathogen clearance, and which collectively can reduce mortality rate.

One significant obstacle to therapy presented by these diseases is the associated cytokine storm. Leng et al. have suggested the potential use of MSCs in mitigating its effects and to this end, have conducted an early phase 7 patient study. In that study, after only three weeks of MSC transplantation, patients’ C plasma levels had dropped significantly [145]. Cao et al. reported that the cytokine storm levels in patients in the ICU had activated immune cells that led to inhibited function of endothelial cells, capillary leakage, and mucous block in the lung and respiratory failure. The administration of MSC led to a decrease in inflammation in severe patients in the ICU, which implies that the use of MSCs helped ICU patients with severe manifestations of coronavirus into recovery [87,145]. Similar to Leng et al., Guo et al. treated 30 patients with UC-MSCs. No adverse effects were observed or attributable from transplantation. However, this study demonstrated the paracrine factors that are known to be secreted by MSCs resulted in the modulation and downregulation of the cytokine storm and assisted in the restoration of oxygenation levels. This suggests that UC-MSCs could improve the lung environment and function and the modulation of pro-inflammatory cytokines [146]. In a study performed by Ercelen et al., MSCs were transplanted into patients suffering from severe COVID-19. They demonstrated that two clinically severe patients did not require intubation after transplantation. Not only were adverse effects not observed but also all patients were discarded from the ICU 1–2 weeks after transplantation. One patient had died from complications not related to transplantation [147]. In a similar study conducted by Sengupta et al., patients were administered the bone marrow-derived exosome product ‘ExoFlo’ intravenously. They demonstrated the reversal of hypoxia, immune reconstitution and, again, the downregulation of the cytokine storm [105]. Collectively these studies prove that MSCs, MSC-CM, and exosomes are a definite asset treating COVID-19 in a clinical setting.

One critical issue is that the appropriate dose for administration has not yet been established. From the present clinical trials, the dosages range from 0.5 × 10^6^ cells kg/body weight to 1 × 10^8^ cells kg/body weight. In addition, the vast majority of these clinical trials administer cells intravenously, which could result in a thrombogenic deposition if not cautious [148]. A limited number of clinical trials have demonstrated exosomes’ ability to reduce lung inflammation and pathological impairment that stem from various types of lung injury. Intravenous administration of exosomes may result in aggregation or clumping in the injured microcirculation, and there is a higher risk of mutagenicity and oncogenicity. This issue does not arise when using nebulizers, specifically nebulized MSCs-Exo [149]. Previously, this review discussed the use of aerosolized MSCs due to their ability to target the lungs in ways intravenous administration is unable to. Aerosol MSC-based therapy is a novel strategy that is capable of enhancing reparative and regenerative processes in both chronic and acute lung injuries [149]. Researchers and biomedical disciplines are beginning to investigate the tolerance of aerosol inhalation of mesenchymal stem cell exosomes (see Table 4).

## 5. Conclusions

The primary objective of this review was to shed light on the potential benefits of MSC-based therapies for respiratory and pulmonary diseases and infections and the potential benefits of aerosolized and nebulized MSC-based therapies. Cell-based therapies, notably MSC-based therapies, are at the forefront of treatment of lung diseases due to their anti-inflammatory, regenerative, and immunomodulatory properties. The pleiotropic effects of MSCs and their potential to be enhanced by preconditioning and potentiation methods make them a promising form of therapy. However, preconditioning will have to be established prior to being used clinically. In addition, due to the possibilities of tumor and emboli formation that can result from the administration of MSCs, the EVs, exosomes and MSC secretome may be a better option. Despite the issues that revolve around MSC, they could be an optimum solution in treating various and complex pathologies of lung diseases.

The need for more efficient administration routes is clearly supported by the studies discussed throughout this paper. Even while proven successful in many cases, there are several critical issues that revolve around the use of IV administration that can be avoided using inhalation, aerosolization, or nebulization technology. Studies suggest that aerosolized MSCs, EVs, and MSC-CM can deliver cells directly to the site of injury. Aerosolization maintains high efficacy and high distribution of cells in the lung and also a minimized risk of cell loss and morphological changes that may occur when administered via intravenous, intratracheal, and intranasal administration. They have also demonstrated their capability to be faster acting than other various routes. However, extensive research should be carried out on various cell therapies and their compatibility with different nebulizers before this strategy can be used in a clinical setting.

Finally, for these therapies to become available commercially, they will need to undergo regulatory approval processes, very likely in combination with specific delivery devices for reliable and reproducible dosing. A recent review covers this area in greater detail, and discusses the likely US and EU regulatory frameworks for the ATMP/delivery device combinations [150].

## Figures and Tables

**Table 1:** Mesenchymal stem cell (MSC)-based therapy clinical trials for ARDS (acute respiratory distress syndrome).

NCT No.	Title	Status	Company Name	Disease/Conditions	Route of Administration	Intervention/Mechanism Target	Results	Phase
NCT01775774	Human MSCs for acute respiratory distress syndrome	Completed	University of California	ARDS	Intravenous	Participants received 1 × 10^6^, 5 × 10^6^ and 10 × 10^6^ cells/kg body weight of allogenic BM-HMSCs	A single dose of MSCs demonstrated to be safe and was tolerated well	1
NCT02611609	A phase 1 and 2 study to assess multistem therapy in acute respiratory distress syndrome	Completed	Athersys, Inc	ARDS	Intravenous	MultiStem^®^	N/A	1 and 2
NCT03608592	Human Umbilical Cord Mesenchymal stem cells (MSC) therapy in ARDS	Recruiting	Lv Haijin, Sun Yat-sen University	ARDS	Intravenous	60 × 10^6^ UC-MCSs intravenously administered in 2 h	N/A	N/A
NCT01902082	Adipose-derived Mesenchymal stem cells in acute respiratory distress syndrome	Unknown	Shaoxing Second Hospital	ARDS	Intravenous	Participants will receive 1 × 10^6^ Adipose-derived MSCs on day 2.	N/A	1
NCT02804945	Mesenchymal Stem cells (MSCs) for the treatment of Acute Respiratory Distress Syndrome (ARDS) in patients with Malignancies	Completed	Anderson Cancer Center	ARDS	Intravenous infusion “MSCs by vein”	Participants received 3 × 10^6^ intravenously administered on day 1	N/A	1
NCT03042143	Repair of Acute Respiratory Distress Syndrome by Stromal cell Administration (COVID-19) (Realist)	Recruiting	Belfast Health and Social Care Trust	COVID/ARDS	Intravenous	Participants will receive max. tolerated dose of human umbilical cord derived CD362 enriched MSCs given in 30–90 min.	N/A	1 and 2
NCT04347967	Mesenchymal stem cells for the treatment of Acute Respiratory Distress Syndrome (ARDS)	Not recruiting yet	Meridigen Biotech Co. Ltd.	ARDS	Intravenous Infusion	Participants will receive low, medium, and high doses of UMC119-06 given to 3 different set of people	N/A	1
NCT03818854	Mesenchymal Stromal Cells for Acute Respiratory Distress Syndrome (STAT)	Recruiting	University of California	ARDS	Intravenous	Participants will receive 10 × 10^6^ cells given over 60–80 min	N/A	2
NCT04377334	Mesenchymal Stem cells (MSCs) in inflammation-Resolution Programs of Coronavirus Disease 2019 (COVID-19) Induced Acute Respiratory Distress Syndrome (ARDS)	Not Recruiting Yet	University Hospital Tuebingen	ARDS/COVID	Intravenous	Participants will receive allogenic bone marrow derived HMSCs	N/A	2
NCT02444455	Human Umbilical-Cord-Derived Mesenchymal Stem Cell Therapy in Acute Lung Injury (UCMSC-ALI)	Unknown	Affiliated Hospital to Acadmey of Military Medical Sciences	ARDS/ALI	Intravenous	Participants will/would receive 5 × 10^5^ cells kg/body weight of human UC-MCSs on day 2, 7 and 14	N/A	1 and 2
NCT02215811	Treatment of Severe Acute Respiratory Distress syndrome with allogenic bone marrow-derived Mesenchymal Stromal cells	Unknown	Karolinska University	ARDS	N/A	Participants will/would receive biological MSCs	N/A	1
NCT02112500	Mesenchymal stem cell in patients with acute severe respiratory failure (STELLAR)	Unknown	Asan Medical Center	Respiratory Distress Syndrome	Intravenous	Participants will/would receive biological MSCs	N/A	2
2019-002688-89	Phase 1/2 clinical study to assess the feasibility, safety, tolerability, and preliminary efficacy of the administration of HCR040, a drug whose active substance is HC016, allogeneic adipose-derived adult mesenchymal stem cells expanded and pulsed with H_2_O_2_, in patients with acute respiratory distress syndrome. (included patients COVID-19)	Ongoing	Histocell S.L	ARDS	Intravenous	Participants will receive HCR040 (allogenic adipose derived adult mesenchymal stem cells pulsed with H_2_O_2_)	No results available	1 and 2
2020-001505-22	Double-blind, randomized, parallel, placebo-controlled pilot clinical trial, nested in a prospective cohort observational study, for the evaluation of the efficacy and safety of two doses of WJ-MSC in patients with acute respiratory distress syndrome secondary to infection by COVID-19	Ongoing	Banc de Sang I Teixits	ARDS	Intravenous	Participants will receive 2 doses of WJ-MSCs	N/A	1
NCT04447833	Mesenchymal Stromal Cell Therapy for The Treatment of Acute Respiratory Distress Syndrome (ARDS-MSC-205)	Recruiting	Uppsala University	ARDS	Intravenous	1st 3 participants will receive 1 × 10^6^ and next 6 participants will receive 2 × 10^6^ of allogenic BM-MSCs	N/A	1
NCT04456361	Use of Mesenchymal Stem Cells in Acute Respiratory Distress Syndrome Caused by COVID-19	Active, not recruiting	Instituto de Medicina Regenerativa	ARDS, Human COVID-19	Intravenous	Participants will receive 1 × 10^8^ dose of Wharton jelly MSCs	N/A	1
NCT03807804	Efficacy and Safety Study of HLCM051 (MultiStem^®^ for Pneumonic Acute Respiratory Distress Syndrome (ONE-BRIDGE)	Recruiting	Healios K.K.	Respiratory Distress Syndrome	Intravenous	One dose of HCLM0S1 consisting of 9.0 × 10^8^ of cells	N/A	2
NCT04371393	MSCs in COVID-19 ARDS	Recruiting	Icahn School of Medicine at Mount Sinai	ARDS, COVID-19	Intravenous	One dose of 2 × 10^6^ MSCs cells/kg body weight	N/A	3
NCT02095444	Using Human Menstrual Blood Cells to Treat Acute Lung Injury Caused by H7N9 Bird Flu Virus Infection	Unknown	S-Evans Biosciences Co., Ltd.	ALI/ARDS and multiple organ failure	Intravenous	Participants will receive 1 dose of 1 × 10^7^ menstrual blood stem cells/kg body weight twice a week for 2 weeks	N/A	1 and 2
NCT04345601	Mesenchymal Stromal Cells for the Treatment of SARS-CoV-2 Induced Acute Respiratory Failure (COVID-19 Disease)	Not recruiting yet	Baylor College of Medicine	ARDS/ COVID-19	Intravenous	Participants will receive 1 × 10^8^ MSCs.	N/A	Early phase 1
NCT04452097	Use of hUC-MSC Product (BX-U001) for the Treatment of COVID-19 With ARDS	Not recruiting yet	Baylx Inc.	ARDS/COVID-19	Intravenous	Participants will receive 1 dose of 0.5 × 10^6^, 1.0 × 10^6^ or 1.5 × 10^6^ cells/kg of body weight	N/A	1
NCT04400032	Cellular Immuno-therapy for COVID-19 Acute Respiratory Distress Syndrome—Vanguard (CIRCA-19)	Not recruiting yet	Ottawa Hospital Research institute	COVID-19, ARDS	Intravenous	75 × 10^6^, 150 × 10^6^ and 270 × 10^6^ BM-MSCs given to 2 sets of groups	N/A	1
NCT04331613	Safety and Efficacy of CAStem for Severe COVID-19 Associated With/Without ARDS	Recruiting	Chinese Academy of Sciences	COVID-19, Acute Respiratory Distress Syndrome, Pneumonia and Acute Lung Injury	Intravenous	3 cohorts with 3 patients will receive 3 × 10^6^, 5 × 10^6^ and 10 × 10^6^	N/A	1 and 2
NCT04390152	Safety and Efficacy of Intravenous Wharton’s jelly derived Mesenchymal stem cells in acute respiratory distress syndrome due to COVID-19	Not recruiting yet	BioXcelleraltor	COVID-19, ARDS	Intravenous	Participants will receive 2 doses of 50 × 10^6^ WJ MSC and hydroxychloroquine, lopinavir or azithromycin and ventilation support	N/A	1 and 2
NCT04345601	Mesenchymal Stromal cells for the treatment of SARS-CoV-2 Induced Acute Respiratory Failure (COVID-19 Disease)	Not recruiting yet	Baylor College of Medicine	COVID-19, ARDS	Intravenous	Participants will be given 1 × 10^8^ MSCs	N/A	1
NCT04390139	Efficacy and safety of Evaluation of Mesenchymal stem cells for the treatment of patients with Respiratory Distress Due to COVID-19 (COVIDMES	Recruiting	Banc de Sang i Teixits	COVID-19, ARDS	Intravenous	Participants will receive 1 × 10^6^ cells/kg body weight W-J MSCs on day 1 and day 3	N/A	1 and 2
NCT04399889	Human Cord Tissue- MSCs for COVID-19	Not recruiting yet	Joanne Kurtzberg, MD	COVID-19, ARDS	Intravenous	Participants will receive hCT-MSCs	N/A	1 and 2
NCT04355728	Use of UC-MSCs for COVID-19 Patients	Recruiting	Camillo Ricordi, University of Miami	COVID-19. ARDS, Acute Lung Injury	Intravenous	Participants will receive 1 × 10^8^ UC-MSCs and standard treatment	N/A	1 and 2
NCT04377334	Mesenchymal Stem Cells (MSCs) in inflammation-resolution programs of Coronavirus Disease 2019 (COVID-19) Induced Acute Respiratory Distress Syndrome (ARDS)	Not recruiting yet	University Hospital Tuebingen	COVID-19, ARDS	Intravenous	Participants will receive allogenic bone marrow-derived human mesenchymal stem cells	N/A	2
NCT04348461	Battle Against COVID-19 Using Mesenchymal Stromal Cells	Not recruiting yet	Instituto de Investigacóin Sanitaria de la Fundación Jieménez Díaz	COVID-19, Respiratory Distress Syndrome	Intravenous	Participants to receive 2 doses of 1.5 × 10^6^ ad-MSCs cells/kg body weight	N/A	2
NCT04367077	MultiStem Administration for COVID-19 Induced ARDS (MACoVIA) (MACoVIA)	Recruiting	Athersys	COVID-19, ARDS	Intravenous	Participants to receive doses of MultiStem	N/A	2 and 3

N/A = Not yet available.

**Table 2:** MSC-based therapy clinical trials for sepsis.

NCT No.	Title	Status	Company Name	Disease/Conditions	Route of Administration	Intervention/Mechanism Target	Results	Phase
NCT02789995	Dysfunctions of human muscle stem cells in Sepsis	Completed	Institut Pasteur	Sepsis	Intravenous Donation	Study of patients with or without sepsis, blood and bone marrow sample and muscle biopsy	N/A	N/A
NCT02421484	Cellular immunotherapy for septic shock: A phase 1 trial (CISS)	Completed	Ottawa Hospital Research Institute	Sepsis	Intravenous	0.3 × 10^6^, 1 × 10^6^ and 3 × 10^6^ cells/kg body weight was administered to participants	Single dose of MSCs demonstrated to be safe and was tolerated well.	1
NCT03369275	Cellular Immunotherapy for Septic Shock (CISS2)	Not yet recruiting	Ottawa Hospital Research Institute	Sepsis, Septic Shock	Intravenous	Participants will receive 3 × 10^8^ BM- HMSCs	N/A	2
NCT01849237	Russian clinical trial of mesenchymal stem cells in patients with septic shock and severe neutropenia	Unknown	National Research center for Hematology	Sepsis	Intravenous	Participants will receive 1–2 × 10^6^ MSC intravenous infusions up to 10 h after septic shock.	N/A	1 & 2
NCT02883803	Treatment of Severe Infections with Mesenchymal Stem Cells (CHOCMSC)	Not yet recruiting	Central Hospital Nancy France	Septic Shock	Intravenous	Participants will receive 1 × 10^6^ cells/kg body weight after 12 h of septic shock	N/A	2
NCT02328612	Randomized, Parallel Group, Placebo Control, Unicentric, Interventional Study to Assess the Effect of Expanded Human Allogeneic Adipose-derived Mesenchymal Adult Stem Cells on the Human Response to Lipopolysaccharide in Human Volunteers (CELLULA)	Completed	Tigenix S.A.U	Sepsis	Intravenous	0.25 × 10^6^, 1 × 10^6^ and 4 × 10^6^ cells/kg body weight were administered to different sets of participants	Intravenous infusion of the cells exhibited anti-inflammatory effects and proved to be safe and efficient	1

N/A = Not yet available.

**Table 3:** MSC based therapy clinical trials for COVID-19.

NCT No.	Title	Status	Company Name	Disease/Conditions	Route of Administration	Intervention/Mechanism Target	Results	Phase
NCT04333368	Cell therapy using Umbilical cord-derived Mesenchymal Stromal Cells in SARS-CoV-2 related ARDS (STROMA-CoV2)	Recruiting	Hopitaux de Paris	COVID-19	Intravenous	Participants will receive 1 × 10^6^ UC-MSCs in 60 min. Via peripheral or central venous line	N/A	1 and 2
NCT04392778	Clinical use of stem cells for the treatment of COVID-19	Recruiting	SBÜ	COVID-19, Pneumonia, Multiple Organ Failure, CoronaVirus Infection	Intravenous	Participants will receive 3 × 10^6^ MSC on Day 0, 3 and 6.	N/A	1 and 2
NCT04252118	Mesenchymal stem cell treatment for pneumonia patients infected with COVID-19	Recruiting	Beijing 302 Hospital	COVID-19	Intravenous	Participants will receive 3 × 10^7^ of MSCs on day 0, 3 and 6	N/A	1
NCT04348435	A randomized, double-blind, placebo-controlled clinical trial to determine the safety and efficacy of hope biosciences allogenic mesenchymal stem cell therapy (HB-adMSCs) to provide protection against COVID-19	Enrolling by Invitation	Hope biosciences	COVID-19	Intravenous	2 × 10^8^, 1 × 10^8^ 0.5 × 10^8^ and 0.1 × 10^8^ Allogenic HB-adMSCs given to 4 different sets participants on day 0, 2, 6, 10, and day 14	N/A	2
NCT04366063	Mesenchymal Stem cell Therapy for SARS-CoV-2 related Acute Respiratory Distress Syndrome	Recruiting	Royan Institute	COVID-19	Intravenous	Participants will receive 2 doses of 100 × 10^6^ MSC at day 0 and 2 and 2 doses of EVs at day 4 and 6	N/A	2 and 3
NCT04288102	Treatment with mesenchymal system cells for severe Coronavirus Disease 2019 (COVID-19)	Recruiting	Beijing 302	COVID-19	Intravenous	Participants will receive 4 × 10^7^ MSCs 3 times a day on day 0, 3 and 6	N/A	2
NCT04349631	A Clinical Trial to Determine the safety and efficacy of Hope Biosciences Autologous Mesenchymal Stem Cell Therapy (HB-adMSCs) to provide protection Against COVID-19	Enrolling by Invitation	Hope biosciences	COVID-19	Intravenous	Participants will receive 5 IV infusions of autologous, AD-MSCs collected and infused and follow up on week 6, 14, 26	N/A	2
NCT04324996	A Phase I/II Study of universal off-the-shelf- NKG2D-ACE2 CAR-NK Cells for Therapy of COVID-19	Recruiting	Chongqing Public Health Medical Center	COVID-19	Intravenous	Participants will receive 1 × 10^8^ cells administered per kilogram of body weight	N/A	1 and 2
NCT04273646	Study of Human Umbilical Cord Mesenchymal Stem cells in the treatment of Severe COVID-19	Not recruiting yet	Wuhan Union Hospital China	COVID-19, 2019 Novel Coronavirus pneumonia	Intravenous	Participants will receive 0.5 × 10^6^ UC-MSCs cells/kg body weight administered on day 1, day 3, day 5, and day 7	N/A	N/A
NCT04397471	A study to collect bone marrow for process development and production of BM-MSC to treat severe COVID-19 Pneumonitis (COMET20d	Not recruiting yet	Cambridge Cellular Therapies Laboratory	COVID-19, Pneumonia	Donation	30–80 mL sample of bone marrow collected from posterior superior iliac crests	N/A	N/A
NCT04382547	Treatment of COVID-19 Associated Pneumonia with Allogenic Pooled Olfactory Mucosa-derived Mesenchymal Stem Cells	Enrolling by Invitation	Institute of Biophysics and Cell Engineering of National Academy of Sciences of Belarus	COVID-19, Pneumonia	Intravenous	Participants will receive standard treatments and also allogenic pooled olfactory mucosa derived MSCs	N/A	1 and 2
NCT04366271	Clinical Trial of Allogenic Mesenchymal Cells from Umbilical Cord Tissue with Patients with COVID-19 (MESCEL-COVID-19)	Recruiting	Hospital Infantil Universitario Nino Jesus, Madrid Spain	COVID-19	Intravenous	Participants will receive 1 infusion of undifferentiated allogenic UC-MSCs	N/A	2
NCT04361942	Treatment of Severe COVID-19 Pneumonia with Allogeneic Mesenchymal Stromal Cells (COVID_MSV) (COVID_MSV)	Recruiting	Red de Terapia Celular	COVID-19, Pneumonia	Intravenous	Participants will receive 1 × 10^6^ MSCs	N/A	2
NCT04346368	Bone Marrow-Derived Mesenchymal Stem cell treatment for severe patients with coronavirus disease 2019 (COVID-19	Not recruiting yet	Guangzhou Institute of Respiratory Disease	COVID-19	Intravenous	Participants will receive 1 × 10^6^ MSCs kg/body weight on day 1	N/A	1 and 2
NCT04416139	Mesenchymal stem cells for acute respiratory distress Syndrome due to COVID-19	Recruiting	Instituto Nacional de Ciencias Medicas y Nutricion Salvador Zubiran	COVID-19	Intravenous	Participants will receive a single dose of 1 × 106 MSCs	N/A	2
NCT04336254	Safety and Efficacy Study of Allogenic Human Dental Pulp Mesenchymal Stem Cells to treat severe COVID-19 Patients	Recruiting	Renmin Hospital of Wuhan University	COVID-19	Intravenous	Participants will receive 3 × 10^7^ human dental pulp stem cells on day 1, day 4 and day 7	N/A	1 and 2
NCT04428801	Autologous Adipose-derived Stem cells (Ad-MSCs) for COVID-19	Not recruiting yet	Celltex Therapeutics Corporation	COVID-19	Intravenous	Participants will receive 2 × 10^8^ Ad-MSCs on day 0, day 3 and day 6	N/A	2
NCT04269525	Umbilical Cord (UC)-Derived Mesenchymal Stem Cells (MSCs) Treatment for the 2019-novel Coronavirus (nCOV) Pneumonia	Recruiting	ZhiYong Peng, Zhongnan Hospital	COVID-19 Pneumonia	Intravenous	Participants will receive UC-MSC infusions and day 1, day 3 and day 5	N/A	2
NCT04429763	Safety and Efficacy of Mesenchymal Stem Cells in the management of severe COVID-19 pneumonia	Not recruiting yet	Trustem	COVID-19	Intravenous	Participants will receive 1 dose of 1 × 10^6^ MSCs	N/A	2
NCT04366323	Clinical Trial to Assess the Safety and Efficacy of Intravenous Administration of Allogeneic Adult Mesenchymal Stem Cells of Expanded Adipose Tissue in Patients with Severe Pneumonia Due to COVID-19	Recruiting	Andalusian Network for design and translation of Advanced Therapies	COVID-19	Intravenous	Participants will receive 8 × 10^7^ allogenic Ad-MSCs	N/A	1 and 2
NCT04352803	Adipose Mesenchymal cells for Abadement of SARS-CoV-2 Respiratory Compromise in COVID-19 Disease	Not recruiting yet	Regeneris Medical	COVID-19, Cytokine Storm	Intravenous	Participants will receive 0.5 × 10^6^ autologous ad-MSCs	N/A	1 and 2
NCT04398303	ACT-20 in Patients with severe COVID-19 Pneumonia	Not recruiting yet	Aspire Health Science	COVID-19 Pneumonia	Intravenous	Participants will receive 1 × 10^6^ allogenic human umbilical derived MSCs	N/A	2
NCT04339660	Clinical Research of Human Mesenchymal Stem Cells in the Treatment of COVID-19 Pneumonia	Recruiting	Puren Hospital Affliated to Wuhan University of Science and Technology	COVID-19	Intravenous	Participants will receive 1 × 10^6^ per kg/bodyweight UC-MSCs	N/A	1
NCT04444271	Mesenchymal Stem Cell Infusion for COVID-19 Infection	Recruiting	Armed Forces Bone Marrow	COVID-19	Intravenous	Participants will receive 1 × 10^6^ cells/kg body weight of MSCs	N/A	1 and 2
2020-001682-36	Double-blind, placebo-controlled phase I/II clinical trial to evaluate the safety and efficacy of allogeneic mesenchymal stem cells (MSV^®^-allo) in acute respiratory failure in patients with COVID-19 pneumonia.	Ongoing	CITOSPIN S.L.	COVID-19	Intravenous	Adult allogeneic stem cell mesenchymal stem cells expanded in suspension	N/A	1
NCT0444520	A Study of Cell Therapy in COVID-19 Subjects with Acute Kidney Injury Who Are Receiving Renal Replacement Therapy	Not recruiting yet	Sentien Biotechnologies, Inc.	COVID-19	Integration	Allogenic SB-101 biologic combination device	N/A	1
NCT04461925	Treatment of Coronavirus COVID-19 Pneumonia (Pathogen SARS-CoV-2) With Cryopreserved Allogeneic P_MMSCs and UC-MMSCs	Recruiting	Institute of Cell Therapy	COVID-19 and Pneumonia	Intravenous	Participants will receive 1 × 10^6^ cells/kg body weight 3 times a day on day 1, 4 and 7	N/A	1 and 2
NCT04445454	Mesenchymal Stromal Cell Therapy for Severe Covid-19 Infection	Recruiting	University of Liege	COVID-19	Intravenous	Participants will receive 3 doses of (1.5)–3.0 × 10^6^/BM-MSC kg/body weight at 3–4 days interval	N/A	1 and 2
NCT04437823	Efficacy of Intravenous Infusions of Stem Cells in the Treatment of COVID-19 Patients	Recruiting	Jinnah Hospital	COVID-19	Intravenous	Participants will receive 5 × 10^5^ of UC-MSCs on day 1,3 and 5	N/A	2
NCT04456361	Use of Mesenchymal Stem Cells in Acute Respiratory Distress Syndrome Caused by COVID-19	Active, not recruiting	Instituto de Medicina Regenerativa	COVID-19	Intravenous	Participants will receive 1 × 10^8^ of Wharton jelly derived UC-MSCs	N/A	2
NCT04457609	Administration of Allogenic UC-MSCs as Adjuvant Therapy for Critically Ill COVID-19 Patients	Recruiting	Indonesia University	COVID-19	Intravenous	Participants will 1st receive standardized treatment oseltamivir and azithromycin and then 1 × 10^6^ UC-MSCs in 100 cc of 0.9% NaCl in 1 h	N/A	2 and 3
NCT04467047	Safety and Feasibility of Allogenic MSC in the Treatment of COVID-19 (COVID19)	Not recruiting yet	Hospital de Clinicas de Porto Alegre	COVID-19	Intravenous	Participants will receive 1 × 10^6^ MSCs	N/A	1
NCT04466098	Multiple Dosing of Mesenchymal Stromal Cells in Patients with ARDS (COVID-19)	Not recruiting yet	Masonic Cancer Center, University of Minnesota	COVID-19	Intravenous	Participants will receive a thawed product consisting of 300 × 10^6^ in DMSO in 1: w/ Dextran 40 + 5% human serum albumin	N/A	2
NCT04486001	Study of Intravenous Administration of Allogeneic Adipose Stem Cells for COVID-19 (CoronaStem1)	Not recruiting yet	Personalized Stem Cells, Inc.	COVD-19	Intravenous	Participants will receive adipose derived stem cells	N/A	1
NCT04490486	Umbilical Cord Tissue (UC) Derived Mesenchymal Stem Cells (MSCs) Versus Placebo to Treat Acute Pulmonary Inflammation Due to COVID-19 (COVID-19)	Not recruiting yet	Joshua M Hare	COVID-19	Intravenous	Participants will receive 1 × 10^8^ UC-MSCS on day 0 and day 3	N/A	1
NCT04456439	Intermediate-size Expanded Access Program (EAP), Mesenchymal Stromal Cells (MSC) for Multisystem Inflammatory Syndrome in Children (MIS-C) Associated with Coronavirus Disease (COVID-19)	Available	Mesoblast International Sàrl	COVID-19	Intravenous	Participants will receive 2 × 10^6^ remestemcel-L within a 5-day period	N/A	N/A
NCT04313322	Treatment of COVID-19 Patients using Wharton Jelly Mesenchymal Stem Cells	Recruiting	Stem Cells Arabia	COVID-19	Intravenous	Participants will receive WJ-MSCs suspended in 25 mL of saline solution	N/A	1
NCT04371601	Safety and effectiveness of mesenchymal stem cells in the treatment of pneumonia of coronavirus disease 2019	Active, Not recruiting	Fuzhou General Hospital	COVID-19 Pneumonia	Intravenous	Participants will first receive standard treatment (oseltamivir and hormones) followed by 1 dose of 1 × 10^6^ cells kg/body weight once daily for 4 days	N/A	1
NCT04362189	Efficacy and Safety of Allogenic HB-adMSCs for the treatment of COVID-19	Recruiting	Hope biosciences	COVID-19	Intravenous	Participants will receive 1 × 10^8^ of HB-adMSCs on day 0, 3, 7 and 10	N/A	2
NCT04384445	Organicell Flow for Patients With COVID-19	Not yet recruiting	Organicell Regenerative Medicine	COVID-19	Intravenous	Participants will receive 1 mL of organicell flow on days 0, 4, and 8	N/a	1 and 2
NCT04341610	ASC Therapy for Patients with Severe Respiratory COVID-19 (ASC COVID-19)	Withdrawn (Not approved by ethical committee)	Rigshospitalet, Denmark	Respiratory tract diseases (COVID-19)	Intravenous	100 million allogenic adipose-derived mesenchymal stem cells in 100 mL saline	N/A	1 and 2
NCT04293692	Therapy for Pneumonia Patients Infected by 2019 Novel Coronavirus	Withdrawn (Patients were transferred to designated hospitals for treatment as needed, the clinical trials cannot be conducted.)	Puren Hospital Affiliated to Wuhan University of Science and Technology	COVID	Intravenous	0.5 × 10^6^ UC-MSCs kg/body weight suspended in 100 mL saline on day 1, day 3, day 5 and day 7	N/A	N/A

N/A= Not yet available.

**Table 4:** Aerosol and aerosolized cell therapy clinical trials.

NCT No.	Title	Status	Company Name	Disease/Conditions	Route of Administration	Intervention/Mechanism Target	Results	Phase
NCT04313647	A Tolerance Clinical Study on Aerosol Inhalation of Mesenchymal Stem Cells Exosomes in Healthy Volunteers	Recruiting	Ruijin Hospital	Safety and Tolerance	Inhalation	2 × 10^8^, 4 × 10^8^, 8 × 10^8^, 16 × 10^8^, 20 × 10^8^ nano vesicles/3 mL to be administered to different sets of participants	N/A	1
NCT04473170	Study Evaluating the Safety and Efficacy of Autologous Non-Hematopoietic Peripheral Blood Stem Cells in COVID-19 (SENTAD-COVID)	Completed	Abu Dhabi Stem Cells Center	COVID-19	Inhalation	Participants were put into groups; group A received NHPBSC through jet nebulization and group B received standard care	N/A	1 and 2
NCT04389385	COVID-19 Specific T cell-derived Exosomes (CSTC-Exo)	Active, Not recruiting yet	TC Erciyes University	COVID-19	Inhalation	Participants will receive specific T cell-derived exosomes (CSTC-Exo) Aerosol inhalation of CSTC-Exo (2.0 × 108 nanovesicles / 3 mL at Day 1, 2, 3, 4 and 5 times daily	N/A	1
NCT04491240	Evaluation of Safety and Efficiency of Method of Exosome Inhalation in SARS-CoV-2 Associated Pneumonia (COVID-19EXO)	Enrolling by Invitation	State-Financed Health Facility “Samara Regional Medical Center Dinasty”	COVID-19	Inhalation	2 sets of participants will receive 0.5–2 × 10^10^ of nanoparticles (exosomes)	N/A	1 and 2
NCT04276987	A pilot clinical study on inhalation of mesenchymal stem cells exosomes treating severe novel coronavirus	Not recruiting yet	Ruijin Hospital	COVID-19	Inhalation	Participants will receive MSC-derived exosomes 5 times, aerosol inhalations of MSC-derived exosomes (2.0 × 10^8^ nano vesicles/3 mL at Days 1, 2, 3, 4 and 5)	N/A	1

N/A = Not yet available.
